# Resistance Response of Chilli (*Capsicum annuum* L.) F_1_ to *Fusarium oxysporum* Involves Expression of the *CaChi*2 Gene

**DOI:** 10.21315/tlsr2018.29.2.3

**Published:** 2018-07-06

**Authors:** Rejeki Siti Ferniah, Rina Sri Kasiamdari, Achmadi Priyatmojo, Budi Setiadi Daryono

**Affiliations:** 1 Department of Biology, Universitas Diponegoro, Jl. Prof. Sudharto SH Tembalang Semarang 50275, Indonesia; 2 Faculty of Biology, Universitas Gadjah Mada, Jl Teknika Selatan Sekip Utara Yogyakarta 55281, Indonesia; 3 Faculty of Agriculture, Universitas Gadjah Mada, Jl Flora Bulaksumur Yogyakarta 55281, Indonesia

**Keywords:** Chitinase, Cross-Breeding, Disease Severity Index (DSI), qRT-PCR

## Abstract

Cross-breeding is a method of producing progeny with better resistance to pathogens. Resistance to pathogens usually involves pathogenesis-related (PR) proteins. Class II chitinase is an example of a defensive PR protein in plants. The class II chitinase in chilli is coded by the *CaChi*2 gene. In this study, we crossed susceptible with resistant chilli cultivars, analysed the F_1_ resistance response against pathogenic *F. oxysporum*, and analysed the level of *CaChi*2 gene expression in the F_1_. Data were collected using disease severity index (DSI) determination and gene expression analysis by qRT-PCR (quantitative Reverse Transcriptase Polymerase Chain Reaction). Results showed that the DSI of F_1_ was not significantly different from the resistant ancestor. The relative *CaChi*2 expression level of F_1_ was higher than the susceptible ancestor but not significantly different from the resistant ancestor. We concluded that the F_1_ can be categorised as resistant to *F. oxysporum*, and the *CaChi*2 gene is involved in the molecular defense response.

## INTRODUCTION

Chilli (*Capsicum annuum* L.) is an important commodity in Indonesia, but its yield has decreased due to pathogen infections. Fusarium wilt caused by *F. oxysporum* is a common pathogen in chilli plantations in Thailand and a potential pathogen in India, China and Indonesia (Ali 2006). Fusarium wilt in Thailand can destroy chilli plantations, with disease severity of 26%–79% (Wongpia & Lomthaisong 2010). The pathogen enters the plant through the roots, but the effects can be seen as wilting of leaves, vein clearing in younger leaflets, epinasty, stunting, and yellowing of older leaves (Agrios 2005). *F. oxysporum* is host-specific, so chilli pathogens cannot infect other plants. However, spores of the pathogen survive in the soil and can infect chilli plants in the future.

The Indonesian Convention for Plant Protection has an integrated plant disease management system that includes plantations of resistant cultivars. Resistant cultivar development is an effective and efficient method in plant cultivation. The resistant cultivars can be individually screened to produce a pure grove, or they can be produced through hybridisation of a resistant and a susceptible cultivar, to make resistant progeny with good characters. Semangun (2006) stated that local cultivars have good pathogen defense, so they are used as parental plants in cultivation. Cross-breeding between resistant and susceptible cultivars would increase the F_1_ resistance compared to the susceptible parents.

One defense mechanism in plants is the production of pathogenesis-related (PR) proteins. For example, chitinase is a PR protein produced by plants to respond to pathogenic infection. Plant chitinase can degrade chitin in the pathogen’s cell wall or enhance the chitinase gene expression in many plant tissues. Plant chitinase is expressed in cabbage, pea, strawberry and tobacco (Amian *et al*. 2011; Ntui *et al*. 2011; Ahmed *et al*. 2012). Chitinase expression increases in resistant plants and inhibits pathogen infection in grape and acacia (Vasanthaiah *et al*. 2010; Rushanaedy *et al*. 2012).

There are many classes of chitinase gene based upon structure, including class I, II, III, IV and V. Class II chitinase has about 250 amino acids with no cysteine-rich domain (Collinge *et al*. 1993; Buhler *et al*. 2007). One of the class II chitinase genes is the *CaChi*2 gene, which is found in *Capsicum annuum*. The *CaChi*2 gene is expressed highly in response to *Phytophthora capsici* and *Colletotrichum coccodes* (Hong & Hwang 2005). *CaChi*2 expression also increases in resistant chilli as a response to *F. oxysporum* (Ferniah *et al*. 2015). However, *CaChi*2 expression levels in F_1_ progeny of the resistant and susceptible crosses are not yet known.

The objectives of this research were to analyse the resistance response of F_1_ from cross-breeding a susceptible chilli cultivar with a resistant cultivar and to analyse the *CaChi*2 expression of the F_1_ progenies.

## MATERIALS AND METHODS

Pathogenic *F. oxysporum* was isolated from Fusarium wilting chilli plants, which was found in Tawangmangu, Central Java, Indonesia (Ferniah *et al*. 2014). The two cultivars of *C. annuum* used in this research were Branang, a resistant cultivar, and Lembang-1, a cultivar susceptible to pathogenic *F. oxysporum* (Ferniah *et al*. 2015).

### Cross-Breeding

Branang and Lembang-1 were cross-bred in a greenhouse. Male flowers were complete blooming flowers but female flowers were selected as the bud flowers. Pollen was collected from male flowers and then wiped onto the stigmas of female flowers. Crossing of chilli plants was performed between 6:00 pm and 8:00 pm.

Hybrids of male (♂) Lembang-1 and female (♀) Branang were named as LB, and hybrids of ♂ Branang and ♀ Lembang-1 were named as BL genotypes. Both LB and BL were grown in a greenhouse with watering every day.

### Fungal Inoculation and Disease Severity Index

*F. oxysporum* was grown in potato dextrose broth (PDB) for four days. Conidia densities were calculated using a hemocytometer and were adjusted to 10^6^ conidia/mL. The conidia were inoculated on 30-day-old chilli plants by root dip method (Herman & Perl-Treves 2007; Karimi *et al.* 2010). Disease symptoms were observed every other day post-inoculation for 25 dpi. Symptoms were recorded using the following system: Score 0 = no symptom, 1 = lower height compared to control, 2 = lower height and chlorosis, 3 = 10% chlorosis and/or 10% wilting, 4 = 11%–25% wilting, 5 = 26%–50% wilting, 6 = 51%–100% wilting and dead. The disease severity index (DSI) was determined with the following equation (Wongpia & Lomthaisong 2010):

$$\text{DSI}=\sum \frac{\left(\text{Disease severity scale}\times \text{number of plants in each scale}\right)}{\left(\text{Highest numerical scale index}\times \text{total number of plants}\right)}\times 100$$

Based on their DSI, plants were categorised as highly resistant (HR) if 0% < DSI ≤ 2%, resistant (R) if 2% < DSI ≤ 10%, susceptible (S) if 10% < DSI ≤ 30%, and highly susceptible (HS) if 30% < DSI ≤ 100% (modified from Nsabiyera *et al*. 2012).

### Measuring *CaChi*2 Expression by qRT-PCR

RNA isolation and gene expression analyses of 5 plants from F_1_ and 5 plants from each of the parental plants were completed at 0, 4, 15 and 25 dpi. RNA was isolated from 30–50 mg of chilli leaves using the Plant Total RNA Mini Kit (Geneaid, UK) according to the protocol. The quantity and quality of the RNA were measured using a NanoVue spectrophotometer (GE Healthcare, UK).

The RNA was amplified by one-step qRT-PCR using the KAPA SYBR FAST One-Step qRT-PCR Kit (KAPA Biosystems, Africa) according to the protocol. The 18S rRNA gene was used as the *normalized gene* (Norm) because its expression level was stable between in treated and non-treated plants (*unpublished*). The *CaChi2* gene as the *gene of interest* (GOI) was amplified based on GeneBank database (Hong & Hwang 2005). Primer pairs for qRT-PCR were 18n for the Norm and c2p for the GOI, which were designed using Primer3Plus software (https://primer3plus.com). Sequence of the 18n forward primer was 5′-GGGCGACTAATGAACCCCAA-3′ with reverse sequence 5′-AAGCACACGTCCGCTTGATA-3′ and 103 bp PCR product. The c2p forward primer was 5′-CACCAGCAGATAGGTCAGCA-3′ with reverse sequence 5′-TCCAGTGGGAACATTCAACA-3′ and 158 bp PCR product. PCR was performed with a Rotor-Gene Q 5-Plex (Qiagen, Germany). One-step qRT-PCR was programmed for 5 min at 42°C for cDNA synthesis, 3 min at 95°C for inactivation of RT, and 40 cycles of amplification with 3 s at 95°C for denaturation, 20 s at 55°C for annealing, and 30 s at 72°C for extension.

Chitinase gene expression in the treated plants was compared to expression in the control plants. Analyses of the expression was based on the Ct value of the GOI and normalised gene. Ct is defined as the number of cycles required for the fluorescent signal to cross the threshold. The Ct values were analysed by the delta-delta Ct method (Livak & Schmittgen 2001) with Rotor-Gene Q Software version 2.1.1 (Qiagen, Germany).

## RESULT AND DISCUSSION

Cross-breeding between Branang and Lembang-1 produced many genotypes, as described in Table 1 and Table 2. Not all of the seeds were able to grow, and many seeds grew dwarfs or died after few days.

Hybridisation of male Lembang-1 with female Branang generated 13 pollinations from 52 crosses, produced 9 mature fruits contained 154 viable seeds. It produced 89 plants named as LB genotypes. Hybridisation of male Branang with female Lembang-1 only generated 5 pollinations from 98 crosses, produced 3 mature fruits contained 38 viable seeds, and then produced 20 plants named as BL genotypes.

The BL genotypes grew dwarfs, and most of them died before treatment. Fungal treatment was applied to 30-days-old LB genotypes by the root dip method, and treated plants were then compared to the parental plants. The DSI of F_1_ (5.7%) was not different significantly from Branang (2%), so the F_1_ was determined to be a resistant cultivar (Table 3).

**Figure 1:**
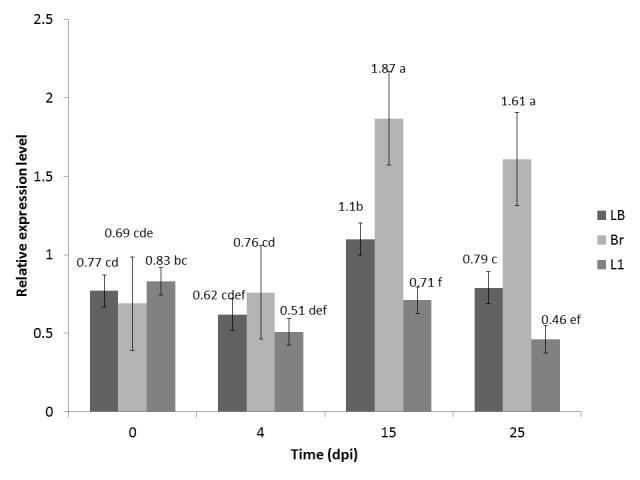
Relative expression levels of *CaChi*2 after *F. oxysporum* infection in F_1_ hybrid (LB) and ancestors Branang (Br) and Lembang-1 (L1). Note: Values in this bar with the same letter are not statistically significantly different at *P* < 0.05.

Expression analysis of the *CaChi*2 gene in F_1_ is shown in Fig. 1. Molecular response of the chilli plants in this study has shown that the more resistant plants expressed higher levels of *CaChi*2 than the susceptible plants. The relative expression levels of *CaChi*2 gene in the resistant plants (Br and LB) were higher than those of the susceptible plant Lembang-1 after 4 dpi. Plants need time to increase their relative expression level as a response to pathogen attack. The time required ranges from a few minutes to several hours after inoculation, and the expression can be maintained for few days to inhibit pathogen sporulation (Schickler & Chet 1997). Resistant cultivars of wheat needed 72 hpi (hours post-inoculation) to enhance their expression level after *Puccinia striiformes* attack (Mohammadi *et al*. 2001).

Plants have developed many mechanisms to resist pathogen attack, including physical defense, chemical defense, and behavioural avoidance of the pathogen (Agrios 2005). Chitinase is a chemical defense that is coded for by chitinase genes, one of which is *CaChi*2 in *Capsicum annuum*. Chitinase acts as a PR protein by degrading the fungal cell wall directly (Hong & Hwang 2005) or by releasing an endogenous elicitor that stimulates systemic acquired resistance (SAR) in plants (Vallad & Goodman 2004). Results in this study showed that expression of *CaChi*2 in the context of SAR occurred in the leaves of the chilli plants although leaves are not the target tissues (root of chilli plants) of pathogen attack. This was demonstrated by the differences in gene expression level of the leaves between resistant and susceptible plants. The expression profile of resistant and susceptible plants in this study confirms the findings of previous research (Ferniah *et al*. 2015).

Hybridisation of plants is aimed at finding new and better properties, including resistance to pathogens. Cross-breeding between Branang and Lembang-1 generated F_1_ progenies that had resistance to *F. oxysporum*, at similar levels to Branang. The resistance of F_1_ was also noted by Keller *et al*. (2000) that plant resistance is usually a dominant phenotype. Inheritance of plant resistance could be monogenic, oligogenic or polygenic (Keller *et al*. 2000; Chahal & Gosal 2002).

Relative expression level of the *CaChi*2 gene was higher in F_1_ than in its susceptible ancestor. Based on the relative expression level, the F_1_ could be categorised as an incompatible or resistant cultivar to *F. oxysporum*. According to Su *et al*. (2015), many chitinase gene expression levels were increased in incompatible sugarcane cultivars in response to sugarcane smut (*Sporisorium scitamineum*). The increasing level of chitinase expression varied over 24–168 hpi (hours post inoculation).

Analyses of F_1_ plant defense and relative expression level of the *CaChi*2 gene showed that the plant defense was affected by the *CaChi*2 gene. Previous studies have demonstrated that the chitinase gene must work synergistically with another gene to express plant defense against fungal pathogen. Hong and Hwang (2005) stated that the chitinase gene must be expressed with the class I pathogenesis-related protein *CaBPR*1 and glucanase (*CaBGlu*) to activate chilli defense against pathogenic fungi. Research on the pea (Amian *et al*. 2011) found that it required chitinase and glucanase genes to convey plant defense. Most recently, research on chilli’s chitinase gene showed that chitinase expression increases in response to *Xanthomonas campestris* pv. *vesicatoria* (*Xcv*) but needs to interact with a cytoplasmic kinase protein (PIK-1) to give rise to defense. The main resistance gene involved in plant defense is cytoplasmic kinase protein, while the chitinase gene is an enhancer (Kim *et al*. 2015).

Based on the DSI, cross-breeding between resistant and susceptible cultivars can produce a resistant cultivar in F_1_. The molecular response of F_1_ was to increase the *CaChi*2 expression level to inhibit an *F. oxysporum* pathogen attack. This study concludes that the F_1_ can be categorised as resistant to *F. oxysporum*, and the *CaChi*2 gene is involved in the molecular defence response.

## Figures and Tables

**Table 1:** Genotype hybrids of Branang and Lembang-1 chilli cultivars.

Type of ♂ × ♀	Number of crosses	Pollinations	Mature fruits	Seeds
♂ L1 × ♀ Br	52	13	9	154
♂ Br × ♀ L1	98	5	2	38

**Table 2:** Seed viabilites of genotype hybrids of Branang and Lembang-1 chilli cultivars.

Genotype	∑ seeds	Viable seeds	% viability	Note
LB1	15	6	40	
LB2	26	21	81	
LB3	30	11	37	
LB4	6	4	67	
LB5	20	17	85	
LB6	10	6	60	
LB7	10	0	0	Damaged seed
LB8	19	15	79	
LB9	18	9	50	
∑ LB	154	89		
Ẋ LB	17.1	9.9	55.3	
BL1	14	8	57	
BL2	24	12	50	
∑ BL	38	20		
Ẋ BL	19	10	53.6	

*Notes*: LB: ♂ Lembang-1 × ♀ Branang, BL: ♂Branang × ♀ Lembang-1

**Table 3:** Comparison of F_1_ and ancestor (P) Disease Severity Index (DSI) in response to *F. oxysporum* infection at 15 dpi (days post-inoculation).

Cultivar	DSI (%)*	Resistance
LB (F_1_)	5.7 b	Resistant
Lembang-1 (P)	17 a	Susceptible
Branang (P)	2 b	Resistant

* Values in this column with the same letter are not statistically significantly different at *P* < 0.05.
